# Capturing mechanisms of change: Weekly covariation in anger regulation, hostile intent attribution, and children's aggression

**DOI:** 10.1002/ab.22019

**Published:** 2022-01-20

**Authors:** Sophie C. Alsem, Janna Keulen, Esmée E. Verhulp, Anouk van Dijk, Bram O. De Castro

**Affiliations:** ^1^ Department of Developmental Psychology Utrecht University Utrecht The Netherlands; ^2^ Research Institute of Child Development and Education University of Amsterdam Amsterdam The Netherlands

**Keywords:** childhood aggression, diary report, emotion regulation, hostile intent attribution, within‐person analyses

## Abstract

Interventions for children's aggression typically target assumed underlying mechanisms, such as anger regulation and hostile intent attribution. The expectation here is that targeting these mechanisms will result in *within‐person* changes in aggression. However, evidence for these mechanisms is mostly based on *between‐person* analyses. We, therefore, examined whether within‐person changes in adaptive anger regulation and hostile intent attribution covaried with within‐person changes in children's aggression. Children (*N* = 223; age 7–12; 46% boys) filled out four weekly report measures to assess adaptive anger regulation, hostile intent attribution, and aggression. The psychometric properties of these novel measures were adequate. Results of multi‐level analyses revealed within‐person effects: weekly changes in adaptive anger regulation and hostile intent attribution covaried with changes in children's aggression. This corresponded with between‐person findings on the same data: children with lower levels of adaptive anger regulation and higher levels of hostile intent attribution reported more aggression than other children. These findings support the idea that targeting anger regulation and hostile intent attribution in interventions may lead to changes in individual children's aggression.

## 1 INTRODUCTION

1.1

1.1.1

Aggressive behavior problems in children are among the most common reasons for referral to mental health care (Lochman & Matthys, 2017; Merikangas et al., 2009). Left untreated, aggressive behavior problems are persistent and relatively stable over time (Burks et al., 2001; Girard et al., 2019; Jester et al., 2008), predicting later delinquency, substance abuse, lower academic achievement, disturbances in relationships with peers, and high costs to society (Evans et al., 2021; Foster et al., 2005; Loeber & Farrington, 2000; Stipek & Miles, 2008). Interventions for children's aggression typically target assumed underlying mechanisms, such as anger regulation and hostile intent attribution (Bookhout et al., 2017). The expectation here is that targeting these mechanisms will result in *within‐person* changes in aggression. However, evidence for the associations between these mechanisms and children's aggression consists almost exclusively of findings from *between‐person* analyses (e.g., Crick & Dodge, 1996; De Castro et al., 2005). These between‐person analyses may reveal, for example, that, on average, children who make more hostile intent attributions, become more aggressive over time compared to children who make less hostile intent attributions (i.e., interindividual differences). However, these findings will not reveal whether within‐person changes in children's hostile intent attribution covary with changes in their own aggression (i.e., intraindividual processes). To truly understand mechanisms of change, we need research that examines within‐person change instead of between‐person differences. Our aim of the present study was to examine within‐person covariation in anger regulation, hostile intent attribution, and children's aggression.

Within‐person studies are an important next step in clinical psychology research. Although between‐person evidence is valuable to identify variables that can be targeted in interventions, we cannot conclude that associations found in between‐person analyses are similar to within‐person associations (Burke & Loeber, 2016; Kazdin, 2011). In fact, previous studies have shown that conflating the two can lead to biased results and potentially incorrect conclusions (Berry & Willoughby, 2017; Hoffman & Stawski, 2009). This issue is less abstract than it may seem. Consider the relation between speed of typing and number of typos (Litschge et al., 2010)—even though some people−such as typists−will type faster and make less typos than others (i.e., a *negative* between‐person association), they will also make more mistakes when they type faster (i.e., a *positive* within‐person association; Hamaker, 2012). Another illustrative example comes from developmental psychology research. In one study, between‐person analyses showed that adolescents who were more secretive than others, also perceived *more* privacy invasion by their parents. In contrast, within‐person analyses showed that when an adolescent became more secretive, parental privacy invasion actually *decreased* (Dietvorst et al., 2018). These examples show that, at least in some cases, within‐ and between‐person analyses can yield opposite conclusions. This has important implications for intervention research, where targeting mechanisms based on between‐person findings might inadvertently cause iatrogenic effects.

Many interventions for children's aggression are based on between‐person findings. The present study, therefore, seeks to investigate within‐person associations for two frequently targeted mechanisms of change in interventions for children's aggression: anger regulation and hostile intent attribution (Bookhout et al., 2017). According to the social information processing model, both anger regulation and hostile intent attribution should predict within‐person changes in aggressive behavior (Crick & Dodge, 1994; Lemerise & Arsenio, 2000). This model assumes that children process social information in ordered steps (e.g., encoding, interpretation, goal selection, response generation), resulting in behavioral response. However, empirical research examining anger regulation and hostile intent attribution as predictors of aggression has predominantly used between‐person analyses, such as regression analyses or cross‐lagged panel models at the group level (Hamaker et al., 2015). From these studies, we know that children with higher levels of aggressive behavior also have more difficulties regulating their anger and frustration than other children (Eisenberg et al., 2005; Rothbart et al., 1994), have a limited repertoire of adaptive anger regulation strategies (De Castro et al., 2005; Roberton et al., 2012; Röll et al., 2012), and displayed emotion regulation problems already earlier in their development (Röll et al., 2012). Moreover, children with aggressive behavior problems display a stronger tendency to interpret ambiguously intended social behavior as stemming from hostile intent (Crick & Dodge, 1996; Verhoef et al., 2019), and both experimental and longitudinal research have shown that hostile intent attribution triggers and predicts children's aggression (De Castro et al., 2003; Dodge et al., 2015). Although this body of between‐person evidence is substantive, it is not enough to support these constructs as mechanisms of change in interventions (Hamaker et al., 2015). Only within‐person analyses can inform us whether changes in children's anger regulation and hostile intent attribution will indeed coincide with changes in their aggression.

Research on within‐person associations requires that data are collected at multiple timepoints from multiple individuals (Curran & Bauer, 2011). An appropriate approach for this goal is diary report methods (Bolger et al., 2003; Esposito et al., 2005), which are used to study individuals' behavior on repeated measurements over a predefined period (ranging from days to months; Lischetzke, 2014). Clinical researchers, for instance, have used diary reports to assess weekly changes in children's well‐being, such as the Brief Problem Checklist and the Child Outcome Rating Scale (Casey et al., 2020; Weisz et al., 2012). This approach seems particularly relevant for the study of anger regulation and hostile intent attribution since short‐term variability in these constructs is found to be high. Anger regulation varies over days and situations (Colasante et al., 2016; McMahon & Naragon‐Gainey, 2019) and hostile intent attribution may vary within children depending on the moment and context (De Castro et al., 2003). We, therefore, developed weekly report measures to assess children's adaptive anger regulation, hostile intent attribution, and aggression on a weekly basis.

Our aim of the present study was to investigate within‐person covariation in adaptive anger regulation, hostile intent attribution, and children's aggression. To this end, we first examined the psychometric properties of our newly developed weekly report measure by testing the internal consistency, convergent validity, and concurrent validity. Second, we investigated our main research question: whether within‐person changes in adaptive anger regulation and hostile intent attribution covaried with within‐person changes in aggression—mirroring the between‐person findings of previous research. We used multi‐level analyses to test whether children would report higher levels of aggression in weeks when they reported lower levels of adaptive anger regulation and higher levels of hostile intent attribution. Third, we examined whether our within‐person findings would correspond with between‐person findings with the same data, expecting that children with lower levels of adaptive anger regulation and higher levels of hostile intent attribution, reported more aggression than other children. With our study, we hope to provide more insight into a key assumption underlying current interventions: that changes in anger regulation and hostile intent attribution are related to changes in individual children's aggression.

## 2 METHOD

### 2.1 Participants

Participants were 223 children, 7–12 years of age (54% girls, 46% boys; *M*_age_ = 10.18, *SD* = 1.21). We recruited children from Dutch primary education schools in (sub)urban communities. The schools served mostly middle‐class communities (income inequality in The Netherlands is relatively low; U.S. Central Intelligence Agency, 2018). The six participating schools distributed consent letters to all parents/caregivers of children from Grades 3 to 6. An overview of descriptive statistics for each school separately is provided in Table 1. Active written informed consent was obtained from all parents and twelve‐year‐old children (consent rate 44%). A cinema gift card (€30) was raffled among participating children. The study was approved by the Ethics Review Board of Utrecht University's Faculty of Social and Behavioral Sciences (No. 20‐0204).

**Table 1:** Descriptive statistics of the participants per school

School	*n*	Boys (%)	Girls (%)	*M*_age_	*SD*_age_
1	87	37.9	62.1	9.84	1.21
2	39	51.3	48.7	10.64	1.02
3	14	64.3	35.7	11.67	0.30
4	15	46.7	53.3	11.16	0.84
5	25	56.0	44.0	9.77	1.13
6	43	46.5	53.5	9.87	1.05

### 2.2 Procedure

Data collection took place in children's classrooms during 4 weekly sessions of 5 min (Weeks 1–3) or 30 min (Week 4), which were spaced exactly one week apart. During the first session, research assistants provided children with instructions and a paper booklet containing all study measures. Children filled out the first weekly report, with research assistants present to answer any questions. During the second and third sessions, children filled out the second and third weekly report accompanied by their own teacher. At the fourth session, research assistants asked children to fill out the fourth weekly report, as well as several validated measures assessing anger regulation, hostile intent attribution, and aggression.

### 2.3 Weekly report measures

Based on existing questionnaires, we developed a weekly report to assess adaptive anger regulation, hostile intent attribution, and aggression that we expected to be sensitive to weekly changes. Constructing a short, feasible scale was important because longer or more complicated instruments are not suited for repeated measurements in children (Casey et al., 2020). For the aggression and anger regulation scales, we used similar items as assessed in a recently published intervention trial examining weekly emotion regulation and aggression in adolescents (te Brinke et al., 2021). We conducted a pilot study in another sample of children (*n* = 89) to assess the quality of these items, which led us to replace the anger regulation item ‘This week I was angry' with “This week I managed to do something against my anger” to improve internal consistency. By changing this, internal consistency of the adaptive anger regulation scale increased from low (Cronbach's α ranging from .12 to .39 across weeks) in the pilot study to adequate (α ranging from .67 to .72) in the current study. Internal consistencies were already adequate in the pilot study for the hostile intent attribution scale (α ranging from .65 to .75) and aggression scale (α ranging from .69 to .80).

#### 2.3.1 Adaptive anger regulation

We assessed weekly adaptive anger regulation by asking children to rate three items: “This week I managed to do something against my anger,” “This week I was so angry that I couldn't stop myself,” “This week I was able to calm myself down when I got angry,” on a five‐point scale (1 = *never*; 5 = *very often*). We averaged across items to calculate an adaptive anger regulation score for each week, allowing for missing data in item scores (1.2% missed one item).

#### 2.3.2 Aggression

To assess children's weekly aggression, we asked children to rate three items: “This week I fought with someone,” “This week I kicked or beat someone,” and “This week I called someone names,” on a five‐point scale (1 = *never*; 5 = *very often*). Items were averaged for each week, allowing for missing data in item scores (2.2% missed one item; 0.3% missed two items).

#### 2.3.3 Hostile intent attribution

To assess children's weekly hostile intent attribution, we asked children to rate three items: “This week people were mean to me,” “This week people were nice to me,” and “This week people wanted to bother me.” Children rated the items on a five‐point scale (1 = *never*; 5 = *very often*) and items were averaged for each week, allowing for missing data in item scores (2.2% missed one item; 0.6% missed two items).

### 2.4 Validation measures

#### 2.4.1 Adaptive anger regulation strategies

Children filled out the anger scale of the FEEL‐KJ (Braet et al., 2013), rating their anger regulation strategies over the past month on a 5‐point scale (1 = *almost never*; 5 = *almost always*). Only the adaptive scale was used in this study (14 items; e.g., “When I'm angry I think about how I could solve the problem”). We computed scores as the average across items (Cronbach's α = .88), allowing for missing data in item scores (9.0% missed one item, 0.4% missed three items, and 1.3% missed seven items).

#### 2.4.2 Aggression

We measured children's aggression using the seven‐item Instrument for Reactive and Proactive Aggression (IRPA; Polman et al., 2009). Both children and teachers rated the frequency of children's aggressive behaviors in the past month (e.g., “How often did you/this child kick other children in the past month?”) on a five‐point scale (1 = *did not occur*; 5 = *daily*). We computed aggression scores as the average across items (Cronbach's α_teacher_ = .82 and α_children_ = .73), allowing for missing data in item scores (0.4% of the teachers missed one item; 3.1% of the children missed one item; and 0.4% of the children missed four items).

#### 2.4.3 Hostile intent attribution

Four audiotaped vignettes describing hypothetical, ambiguous peer provocations were used to assess children's hostile intent attribution (adapted from De Castro et al., 2005). Research assistants told children that they would listen to vignettes about daily social events. Children were asked to imagine each story was happening to them. After each story, children filled out two questions: “The other boy did [behavior other boy]. Did he intend to be mean?” and “Did he do this to bother you?” on a 10‐point scale (1 = *not at all*; 10 = *very much*). The eight items were averaged (Cronbach's α = .83), allowing for missing data in item scores (2.2% missed one item and 0.4% missed two items).

### 2.5 Data analyses

We first examined three psychometric properties of our weekly report measures using IBM SPSS Statistics 26. First, we assessed whether internal consistency was adequate, using Cronbach's alpha's (α > .60) and item‐total correlations (*r* > .20; Evers et al., 2010). Second, we examined convergent validity by testing whether the weekly reports were significantly positively associated with validated questionnaires assessing the same construct. Third, we examined concurrent validity by testing whether, in each week, adaptive anger regulation and hostile intent attribution reports were significantly associated with the weekly reports of aggression in the same week.

We examined within‐person and between‐person associations using multilevel analyses in Mplus 8 (Muthén & Muthén, 2007). We took a three‐step approach. First, we executed three random intercept models to assess whether there was significant variance at the within‐ and between‐person level in adaptive anger regulation, hostile intent attribution, and aggression. As significant variance is required to examine within‐person and between‐person associations, this step serves as a prerequisite for the next steps. Second, we executed one model to investigate within‐person associations, entering adaptive anger regulation and hostile intent attribution as predictors for aggression at the within‐person level (i.e., Level 1). We used person‐mean centered variables for these analyses, which we created by subtracting children's own mean score across the four weeks from each of their weekly scores. This allowed us to examine whether lower (than their own average) levels of adaptive anger regulation and higher (than their own average) levels of hostile intent attribution predicted higher levels of aggression within each week. The resulting betas represent the average within‐subject effects across the 4 weeks. Third, we investigated between‐person associations by adding adaptive anger regulation and hostile intent attribution as predictors to the model at the between‐person level (i.e., Level 2). For these analyses, we created grand mean centered variables by subtracting the sample's mean score from children's mean scores across the 4 weeks. This allowed us to examine whether children with lower (than the sample average) levels of adaptive anger regulation and higher (than the sample average) levels of hostile intent attribution also displayed higher levels of aggression. The raw data and analysis code are available at the Open Science Framework (Alsem et al., 2022).

## 3 RESULTS

### 3.1 Preliminary analyses

#### 3.1.1 Missing data

We inspected missingness in our weekly measures. In total, 162 children completed questionnaires in all 4 weeks (72.6%) and almost all children completed questionnaires in at least 3 weeks (97.3%). We compared children that completed all 4 weeks (*n* = 162) with children with at least one missing week (*n* = 61) and found no significant differences in levels of adaptive anger regulation, hostile intent attribution, and aggression. To check for missing data patterns on item level across assessments, we conducted Little's test which produced a normed *χ* ^2^ (*χ* ^2^/*df)* of 1.33, indicating that data were missing at random (Bollen, 1989). We, therefore, used default settings for multilevel data in Mplus to estimate missing data, which is maximum likelihood (MLR; Muthén & Muthén, 2007). Missingness for validation measures was low (3.8%) and was handled using pairwise deletion in SPSS.

#### 3.1.2 Descriptive statistics of the weekly report measures

Descriptive statistics and intercorrelations of children's mean scores across the four weeks were calculated. Children scored on average 4.05 on adaptive anger regulation (*SD* = 0.78; ranging from 1.44 to 5.00), 1.75 on hostile intent attribution (*SD* = 0.62; ranging from 1.00 to 4.33), and 1.58 on aggression (*SD* = 0.59; ranging from 1.00 to 3.89). As expected, children with lower levels of adaptive anger regulation scored higher on hostile intent attribution (*r* = −.37, *p* < .001) and aggression (*r* = −.43, *p* < .001). Children with higher levels of hostile intent attribution also scored higher on aggression (*r* = .53, *p* < .001).

#### 3.1.3 Psychometric properties of the weekly report measures

The internal consistencies of the weekly measures were adequate: Cronbach's α's ranging from .61 to .96, and item‐total correlations ranging from .22 to .72 (see Appendix A in Supporting Information Material S1). The convergent validity of the weekly measures was adequate: Correlations between the weekly reports and validated measures of the same constructs were all significant, with small‐to‐moderate correlations for adaptive anger regulation, small correlations for hostile intent attribution, and large correlations for child‐reported aggression (see Table 2; Cohen, 1988). Last, attesting to the concurrent validity, the weekly reports of aggression were significantly correlated with weekly reports of both adaptive anger regulation (ranging from *r* = −.29 to −.43; all *ps* < .05) and hostile intent attribution in the same week (ranging from *r* = .44 to .53; all *ps* < .05; see Appendix B in Supporting Information Material S1).

**Table 2:** Pearson's correlations of the weekly reports of adaptive anger regulation, hostile intent attribution, and aggression with validated measures assessing the same constructs

	Validation measures			
	Adaptive anger regulation	Hostile intent attribution	Aggression child report	Aggression teacher report
Weekly report week 1	.27**	.24**	.50**	.30**
Weekly report week 2	.30**	.24**	.60**	.32**
Weekly report week 3	.20**	.15*	.63**	.22**
Weekly report week 4	.25**	.17*	.57**	.23**

*Note*: *p < .05; **p < .01.

### 3.2 Main analyses

#### 3.2.1 Within‐person and between‐person variance

There was significant variance at both the within‐ and between‐level in each of the three weekly report variables (all *p* s < .001) of adaptive anger regulation (41.7% within; 58.3% between), hostile intent attribution (45.5% within; 54.5% between), and aggression (37.9% within; 62.1% between). These *within‐person* variances indicate that children fluctuated in their levels of adaptive anger regulation, hostile intent attribution, and aggression over the 4 weeks, whilst the *between‐person* variances indicate that children differed from each other in their average levels of these variables across the four weeks.

#### 3.2.2 Within‐person associations

**Figure 1:**
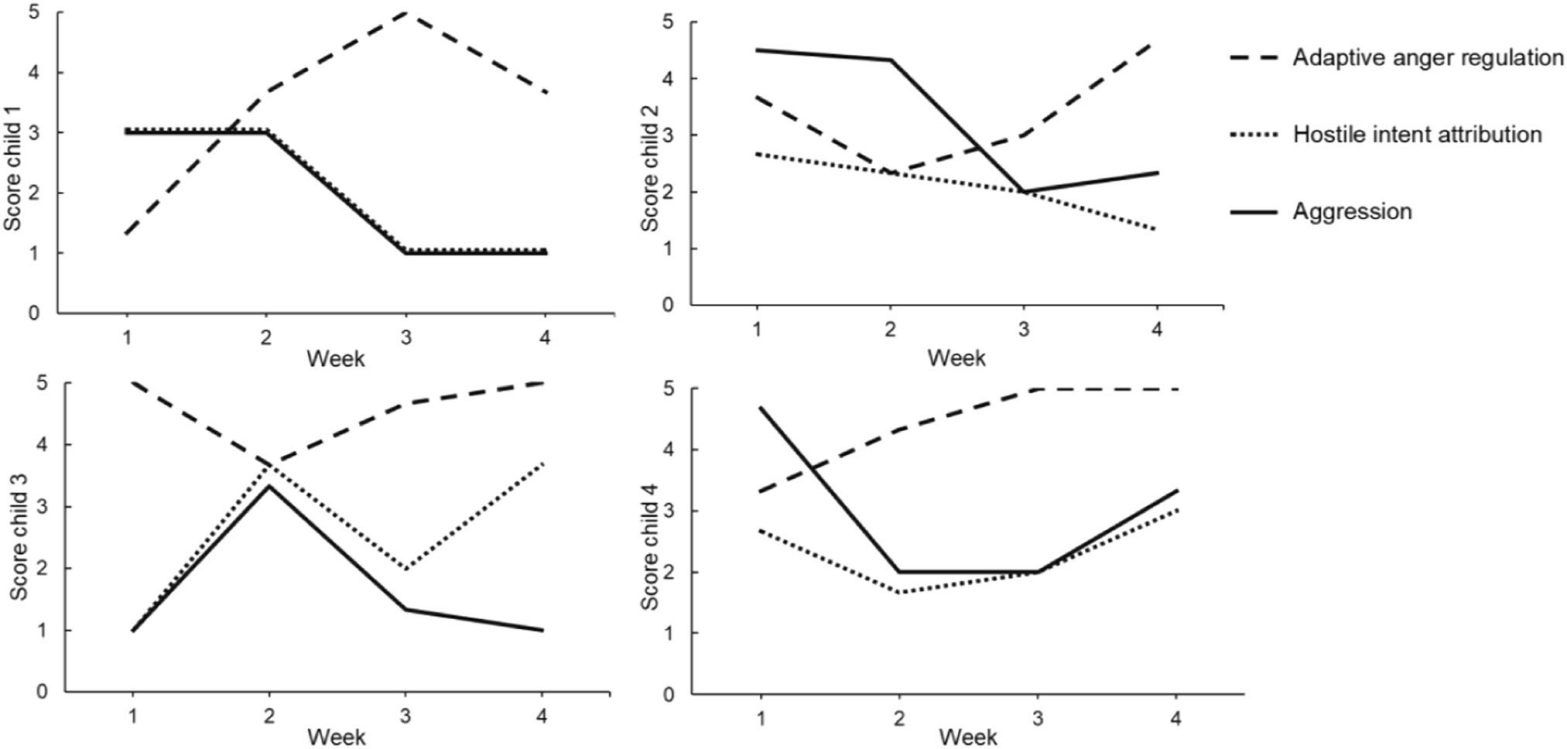
Within‐person change of adaptive anger regulation and hostile intent attribution with aggression in four children with the highest variation in aggression

As expected, we found that within‐person changes in adaptive anger regulation (*B* = −0.11, *SE* = 0.04, β = −.14, *p* = .002) and hostile intent attribution (*B* = 0.28, *SE* = 0.04, β = .30, *p* < .001) were significantly related to within‐person changes in aggression during the 4 weeks (see Table 3). Together, adaptive anger regulation and hostile intent attribution explained 17.2% of variance in aggression at the within‐person level. These findings indicate that children reported more aggression in weeks they reported less adaptive anger regulation and more hostile intent attribution. To illustrate these within‐person effects, Figure 1 presents scores of the four children with the highest variation in aggression.

**Table 3:** Results of the multilevel analyses of the within‐ and between‐person effects of adaptive anger regulation and hostile intent attribution on aggression over 4 weeks

	*B*	*SE*	*β*	*p*
Within person				
Adaptive anger regulation^a^	−0.11	0.04	−.14	.002
Hostile intent attribution^a^	0.28	0.04	.30	<.001
Between person				
Adaptive anger regulation^b^	−0.20	0.06	−.30	<.001
Hostile intent attribution^b^	0.42	0.42	.49	<.001

^a^ Person mean centered.

^b^ Grand mean centered.

#### 3.2.3 Between‐person associations

As expected, we found that adaptive anger regulation (*B* = −0.20, *SE* = 0.06, β = −.30, *p* < .001) and hostile intent attribution (*B* = 0.42, *SE* = 0.06, β = .49, *p* < .001) were significantly related to aggression at the between‐person level across the four weeks (see Table 3). Adaptive anger regulation and hostile intent attribution together explained 38.3% of the variance in aggression at the between‐person level. These findings indicate that children who reported lower levels of adaptive anger regulation and higher levels of hostile intent attribution than others, also showed more aggression than others.

## 4 DISCUSSION

Interventions for children's aggression typically target assumed underlying mechanisms, such as anger regulation and hostile intent attribution. The expectation here is that targeting these mechanisms will result in *within‐person* changes in aggression. However, evidence for these mechanisms is mostly based on between‐person analyses. Therefore, in the present study, we examined within‐person covariation in adaptive anger regulation, hostile intent attribution, and children's aggression over a 4‐week period. We developed weekly report measures to assess adaptive anger regulation, hostile intent attribution, and aggression. These measures showed adequate psychometric quality. Results revealed within‐person associations: weekly changes in adaptive anger regulation and hostile intent attribution covaried with changes in children's aggression. Similar patterns were found at the between‐person level: children who reported lower levels of adaptive anger regulation and higher levels of hostile intent attribution than others, reported more aggression than others over the four weeks.

The present study is the first to replicate findings from earlier between‐person analyses at the within‐person level: changes in adaptive anger regulation and hostile intent attribution were related to changes in aggression *within* children, as depicted in Figure 1 (Crick & Dodge, 1994; De Castro et al., 2005; Verhoef et al., 2019). This finding is crucial for interventions, as it supports the use of anger regulation and hostile intent attribution as mechanisms of change to target in interventions for children's aggression (Bookhout et al., 2017; Hamaker et al., 2015). Future intervention research could build on these findings by targetting children's anger regulation and hostile intent attribution, while assessing within‐person changes in both these mechanisms and children's aggression. That way, researchers may learn whether induced changes in the assumed mechanisms indeed predict decreases in individual children's aggression over the treatment weeks (Kazdin, 2011). Our newly developed weekly report measures may provide an easy and valid tool to do so.

A strength of our study was that it included a relatively large sample of children followed over four weeks, which allowed us to apply a multilevel model. To our knowledge, this study was the first to examine whether within‐person changes in anger regulation and hostile attribution are associated with changes in aggression. In addition, we developed weekly report measures of anger regulation, hostile intent attribution, and aggression, which demonstrated adequate psychometric qualities. If these promising findings are replicated in clinical samples, our weekly report measures could be valuable instruments to monitor mechanisms of change and treatment progress over the course of an intervention.

Our study also had its limitations. First, our main findings relied solely on self‐report. Although self‐reports of children's aggression have been associated with parent‐ and teacher‐report (Achenbach et al., 1987; Marsee et al., 2014), using only self‐reports raises the issue of common method variance. In fact, this issue might have contributed to the high amount of explained variance in aggression that we observed at the between‐person level (38.3%). Future research could build on our findings by studying weekly changes in aggression with reports of multiple informants or observational measures. For hostile intent attribution and anger regulation, however, self‐reports might be the preferred approach since these concepts concern internal processes that may be less visible to parents or teachers than external behavior (Cracco et al., 2015; Crick & Dodge, 1994). Second, the generalizability of our findings is still limited. We assessed only direct aggression, and data were collected in the Netherlands in a relatively well‐functioning community sample of children ages 7–12 years (i.e., children recruited from the regular population with low mean levels of aggression). No data concerning ethnic background were collected. Future research is needed to examine whether our findings generalize to other forms of aggression (e.g., indirect aggression), and to other populations (e.g., children living in other regions, with diverse ethnic backgrounds, or children with aggressive behavior problems). Third, with our analyses we only examined covarying change and were not able to study temporal priority. It would be an interesting avenue for future research to investigate whether fluctuations in anger regulation and hostile intent attribution at one moment temporally predict later changes in aggression. Fourth, the consent rate was relatively low in our study (44%). As consent rates are typically lower in schools serving children from lower socioeconomic backgrounds (Esbensen et al., 2008), future research may study within‐person associations in more diverse samples including children showing higher levels of aggressive behavior.

Our findings open promising directions for future research. First, we showed evidence of spontaneous covarying change over a 4‐week period. An important next step may be to examine within‐person associations when changes in anger regulation and hostile intent attribution are induced in therapy, which may inform us about the causal direction of within‐person changes. Second, at a more fundamental level, it may also be relevant to consider the exact time intervals at which within‐person associations are examined (Keijsers & Van Roekel, 2018). For instance, research has shown that children's anger and aggression covary at a daily basis (Colasante et al., 2016), but conversely, we know that children develop relatively consistent and stable emotion regulation styles and use these across different situations (Roberton et al., 2012). Third, an interesting avenue for future research might be to examine interaction effects of anger regulation and hostile intent attribution on children's day‐by‐day variations in aggression. For instance, it might be that children only become aggressive if they attribute hostile intent at moments when they are not able to regulate anger feelings effectively, for example, because they are tired (Lemerise & Arsenio, 2000).

In conclusion, we have found that within‐person changes in adaptive anger regulation and hostile intent attribution covaried with changes in children's aggression. These findings provide strengthened support for the assumption that targeting anger regulation and hostile intent attribution in interventions may lead to reductions in individual children's aggression. As such, our study may inspire researchers to conduct within‐person studies to investigate assumed mechanisms of change in clinical interventions.

## Supporting information

Supporting information.

## Data Availability

The raw data and analysis code are available at the Open Science Framework (Alsem, Keulen, Verhulp, van Dijk, & De Castro, 2022).
