# Supplementary material for: Capturing mechanisms of change: Weekly covariation in anger regulation, hostile intent attribution, and children's aggression
Source: Aggress Behav. 2022 Jan 20;48(2):232–40. doi: 10.1002/ab.22019 (PMC9306713; doi:10.1002/ab.22019)
Supplement: Supplementary file 1 — Supporting information. [file AB-48-232-s001.pdf]

Appendix A

Cronbach's α and Item-total Correlations of the Weekly Reports of Adaptive Anger Regulation, Hostile Intent Attribution, and Aggression

Measure	Cronbach's α	Min-max of the item-total correlations
Adaptive anger regulation week 1	.67	.27 - .61
Adaptive anger regulation week 2	.69	.22 - .69
Adaptive anger regulation week 3	.70	.24 - .70
Adaptive anger regulation week 4	.72	.28 - .72
Hostile intent attribution week 1	.61	.32 - .58
Hostile intent attribution week 2	.67	.44 - .52
Hostile intent attribution week 3	.66	.34 - .58
Hostile intent attribution week 4	.74	.39 - .68
Aggression week 1	.67	.42 - .61
Aggression week 2	.69	.46 - .61
Aggression week 3	.64	.41 - .56
Aggression week 4	.67	.46 - .55

Note. Min = minimum, max = maximum

Appendix B

Pearson's Correlations of the Weekly Reports and Validated Measures (Assessed in Week 4) of Adaptive Anger Regulation and Hostile Intent Attribution with the Weekly Reports of Aggression in Each of the Four Weeks

	Weekly measure		Validated measures	
	Adaptive anger regulation	Hostile intent attribution	Adaptive anger regulation	Hostile intent attribution
Aggression week 1	-.35**	.44**	-.15*	.16*
Aggression week 2	-.43**	.50**	-.17*	.16*
Aggression week 3	-.35**	.53**	-.13	.11
Aggression week 4	-.29**	.51**	-.16*	.12

** $p < .01$ * $p < .05$

Appendix C

Pearson's Correlations of the Weekly Reports of Adaptive Anger Regulation and Hostile Intent Attribution with the Validated Measures (Assessed in Week 4) of Aggression Reported by Children and Teachers

	Validated measures	
	Child-reported aggression	Teacher-reported aggression
Anger regulation week 1	-.32**	-.13*
Anger regulation week 2	-.29**	-.13
Anger regulation week 3	-.33**	-.06
Anger regulation week 4	-.30**	-.19**
Hostile intent attribution week 1	.23**	.20**
Hostile intent attribution week 2	.25**	.20**
Hostile intent attribution week 3	.41**	.21**
Hostile intent attribution week 4	.36**	.19**

** $p < .01$ * $p < .05$
